# Exploring Class I polyhydroxyalkanoate synthases with broad substrate specificity for polymerization of structurally diverse monomer units

**DOI:** 10.3389/fbioe.2023.1114946

**Published:** 2023-02-21

**Authors:** Ramamoorthi M Sivashankari, Maierwufu Mierzati, Yuki Miyahara, Shoji Mizuno, Christopher T. Nomura, Seiichi Taguchi, Hideki Abe, Takeharu Tsuge

**Affiliations:** ^1^ Department of Materials Science and Engineering, Tokyo Institute of Technology, Yokohama, Japan; ^2^ Department of Biological Sciences, College of Science, University of Idaho, Moscow, ID, United States; ^3^ Graduate School of Science, Technology and Innovation, Kobe University, Kobe, Japan; ^4^ Bioplastic Research Team, RIKEN Center for Sustainable Resource Science, Wako, Japan

**Keywords:** PHA synthases, broad substrate specificities, molecular weight, blast, copolymer

## Abstract

Polyhydroxyalkanoate (PHA) synthases (PhaCs) are key enzymes in PHA polymerization. PhaCs with broad substrate specificity are attractive for synthesizing structurally diverse PHAs. In the PHA family, 3-hydroxybutyrate (3HB)-based copolymers are industrially produced using Class I PhaCs and can be used as practical biodegradable thermoplastics. However, Class I PhaCs with broad substrate specificities are scarce, prompting our search for novel PhaCs. In this study, four new PhaCs from the bacteria *Ferrimonas marina*, *Plesiomonas shigelloides*, *Shewanella pealeana*, and *Vibrio metschnikovii* were selected *via* a homology search against the GenBank database, using the amino acid sequence of *Aeromonas caviae* PHA synthase (PhaC_Ac_), a Class I enzyme with a wide range of substrate specificities, as a template. The four PhaCs were characterized in terms of their polymerization ability and substrate specificity, using *Escherichia coli* as a host for PHA production. All the new PhaCs were able to synthesize P(3HB) in *E. coli* with a high molecular weight, surpassing PhaC_Ac_. The substrate specificity of PhaCs was evaluated by synthesizing 3HB-based copolymers with 3-hydroxyhexanoate, 3-hydroxy-4-methylvalerate, 3-hydroxy-2-methylbutyrate, and 3-hydroxypivalate monomers. Interestingly, PhaC from *P. shigelloides* (PhaC_Ps_) exhibited relatively broad substrate specificity. PhaC_Ps_ was further engineered through site-directed mutagenesis, and the variant resulted in an enzyme with improved polymerization ability and substrate specificity.

## Introduction

The bacterial polyesters polyhydroxyalkanoates (PHAs) are considered excellent bio-based plastics and have been demonstrated to be biodegradable in various environments such as compost, soil, freshwater, and marine water (Suzuki et al., 2021). A myriad of microorganisms can synthesize PHA as an intracellular carbon and energy reserve under stressful conditions (Anderson and Dawes, 1990). Poly[(*R*)-3-hydroxybutyrate], P(3HB), is a major member of the PHA family and has been extensively studied since its discovery in 1926 (Lenz and Marchessault, 2005). Despite these merits, it is still challenging for PHA to compete with petroleum-based plastics because of the inherent flaws in P(3HB). The poor material properties of P(3HB) (Lehrle and Williams, 1994) such as its high crystallinity and narrow processing temperature window have greatly hampered the entry of this polymer into the commercial world. Fortunately, 3HB-based copolymers (Tsuge et al., 2005; Mizuno et al., 2010; Mierzati et al., 2020; Furutate et al., 2021) have been proven to overcome the material property limitations of P(3HB) to a certain extent, and have been used as a remedy for problems related to plastics (Sivashankari and Tsuge, 2021).

PHA synthases are key enzymes involved in PHA polymerization (Sudesh et al., 2000). Based on the substrate specificities and subunit compositions of PHA synthases, they are categorized into four classes (Rehm, 2003). Class I and II PHA synthases are homodimers of the PhaC subunits. Class I PHA synthases, represented by the *Ralstonia eutropha* enzyme, mainly polymerize short chain length (scl)-monomers (C3-C5), whereas Class II PHA synthases, represented by the *Pseudomonas aeruginosa* and *Pseudomonas putida* enzymes, polymerize medium chain length (mcl)-monomers (C6-C14). Class III PHA synthases such as *Allochromatium vinosum* and *Synechocystis* sp. PCC 6803 consists of two heterosubunits (PhaC and PhaE). Class IV PHA synthases, represented by *Bacillus megaterium* and *Bacillus cereus*, are similar to Class III PHA synthases and possess two subunits (PhaC and PhaR). Similar to Class I synthases, Class III and IV PHA synthases preferentially polymerize scl-monomers (C3-C5).

PhaCs with broad substrate specificities are attractive biocatalysts for PHA synthesis because they can naturally copolymerize different monomers to produce polymers with desirable physical properties. PhaC from *Aeromonas caviae* (PhaC_Ac_) can naturally synthesize poly(3HB-*co*-3-hydroxyhexanoate) [P(3HB-*co*-3HHx)] from vegetable oils and fatty acids (Kobayashi et al., 1994; Shimamura et al., 1994; Doi et al., 1995; Tsuge et al., 2007a; Tsuge, 2016), distinguishing it from other Class I PhaCs because it exhibits polymerization activities toward 3HB monomers and mcl 3HHx monomers (Kobayashi et al., 1994). Therefore, PhaC_Ac_ is a marketable biocatalyst to produce P(3HB*-co*-3HHx) copolymers. The potential of PhaC_Ac_ has been fortified through evolutionary engineering with the development of the PhaC_Ac_NSDG variant (Tsuge et al., 2007b). The PhaC_Ac_NSDG variant has amino acid substitutions of asparagine 149 by serine (N149S) and aspartate 171 by glycine (D171G) and was shown to have the ability to synthesize the P(3HB*-co*-3HHx) copolymer with an enhanced 3HHx fraction compared to the wild-type enzyme, as well as recognize and incorporate other monomer units, such as 3-hydroxy-4-methylvalerate (3H4MV) (Tanadchangsaeng et al., 2009) and 3-hydroxy-2-methylbutyrate (3H2MB) (Watanabe et al., 2015). In addition, the molecular weight of P(3HB) synthesized by PhaC_Ac_NSDG was higher than that of the wild-type enzyme (Tsuge et al., 2007b). These properties of PhaC_Ac_NSDG variant are desirable for the development of PHA as an industrial biomaterial, making it a promising biocatalyst.

The partial crystal structures for several PhaCs have been solved (Wittenborn et al., 2016; Chek et al., 2017; Kim et al., 2017; Chek et al., 2020). The differences in the catalytic properties of these enzymes can be possibly due to their different structures (Chek et al., 2019). Although the crystal structure of PhaC_Ac_ has not yet been solved, a basic understanding of the enzymatic capability of PhaC_Ac_ could be elucidated using *in silico* homology modeling (Harada et al., 2021). Additionally, the use of structural information, namely the comparison of the amino acid residues that constitute the substrate-binding pocket of PhaCs, led to the generation of further engineered PhaC_Ac_s (Harada et al., 2021).

PhaC_Ac_ and its PhaC_Ac_NSDG variant are biocatalysts that produce PHA polymers with desirable material properties; however, the number of other naturally occurring PhaCs with broad substrate specificities are limited, hindering the development and commercial mass production of desirable PHAs. Thus, it is necessary to identify other novel PhaCs with broad substrate specificities to enable industrial-scale production of PHA copolymers to completely replace petroleum-based plastics. PhaCs, which can synthesize high-molecular-weight PHA, is essential to produce PHA as practical materials. The currently available PhaC_Ac_ is highly sensitive to ethanol (Hiroe et al., 2015), which is a metabolite of some bacteria, including *Escherichia coli,* and functions as a chain transfer agent to terminate polymerization reactions (Tsuge, 2016), resulting in the synthesis of relatively low-molecular-weight PHA when using *E. coli* as a production host. These low-molecular-weight PHA polymers have less desirable physical properties than their high-molecular-weight counterparts. Despite the unique ability of PhaC_Ac_ to polymerize various monomers, the relatively low molecular weight of PHA produced in recombinant *E. coli* using this enzyme has room for improvement.

In this study, to explore novel PhaCs with high polymerization ability and broad substrate specificity, four new PhaCs were identified by a bioinformatics approach using the PhaC_Ac_ amino acid sequence as a template for a basic local alignment search tool (BLAST) and included PhaCs from the bacteria *Ferrimonas marina, Plesiomonas shigelloides*, *Shewanella pealeana*, and *Vibrio metschnikovii*. PhaC proteins were individually expressed in *E. coli* LSBJ to synthesize P(3HB) and 3HB-based copolymers containing 3HHx, 3H4MV, 3H2MB, and 3-hydroxypivalate (3HPi) units. Furthermore, the effects of mutagenesis on polymerization activity and substrate specificity in the highest-performing PhaC enzyme were investigated.

## Materials and methods

### Bioinformatic analysis

A BLAST-protein (BLASTP) search was performed against the protein sub-sections of the National Center for Biotechnology Information (NCBI) and DNA Data Bank of Japan (DDBJ) databases using the PhaC_Ac_ amino sequence as a template (Accession No. BAA21815) (Altschul et al., 1990). PhaCs with more than 85% similarity index and an identity index of 50%–60% in the BLASTP search were targeted as potential PhaCs with broad substrate specificities. Among the various PhaCs from different organisms that satisfied the criteria in the BLASTP search, four PhaCs were selected based on the diversity of the N-terminal region for further evaluation. Phylogenetic analyses were performed using the maximum likelihood method in MEGA11 (Tamura et al., 2021) and the protein sequences were aligned using ClustalW. This analysis involved six amino acid sequences: PhaC_Ac_, four newly selected PhaCs from BLASTP, and PhaC from *Ralstonia eutropha* (WP_011615085) as an outgroup.

### Bacterial strain and plasmid

Four PhaC amino acid sequences were chosen based on the BLASTP search results. These *phaC* genes were chemically synthesized with optimized codon usage in *E. coli* by Eurofins Genomics Co. Ltd. (Tokyo, Japan) for plasmid construction and evaluation. *E. coli* LSBJ, a *fadB fadJ* double-deletion strain of *E. coli* LS5218 [*fadR601*, *atoC* (Con)] (Tappel et al., 2012a), was used as the host strain for PHA biosynthesis. This strain is an ideal host for non-native PHA production because of its ability to take up a wide variety of substrates to be incorporated into PHA homo- and copolymers, and bench-level scale-up methodologies available for overall production (Tappel et al., 2012b; Levine et al., 2016; Pinto et al., 2016; Fadzil et al., 2018; Furutate et al., 2021; Scheel et al., 2021). A broad-host-range plasmid pBBR1MCS-2 (Kovach et al., 1995) harboring the genes encoding the PhaCs to be evaluated, the *lac* promoter region, the (*R*)-specific enoyl-CoA hydratase gene from *A. caviae* (*phaJ*_Ac_), the 3-ketothiolase gene (*phaA*) from *Ralstonia eutropha* H16, and the acetoacetyl-CoA reductase gene (*phaB*) from *R. eutropha* H16, termed pBBR1-phaCsAB_Re_J_Ac_, was used for the expression of PhaCs (Supplementary Figure S1). For *phaAB* expression, the *R. eutropha pha* promoter and terminator regions were located upstream and downstream of their genes, respectively. To enhance the supply of 3HHx, 3H4MV, and 3H2MB monomers, the plasmid pTTQ-PCT (Furutate et al., 2017) containing the propionyl-CoA transferase (PCT) gene from *Megasphaera elsdenii* (*pct*) (Taguchi et al., 2008) was introduced into the *E. coli* LSBJ strain (Supplementary Figure S1).

### Cell culture conditions

Initially, recombinant *E. coli* LSBJ was incubated overnight at 37°C with reciprocal shaking (160 rpm) in a 50 mL baffle flask containing 20 mL of lysogeny broth (LB) medium as a seed culture. The LB medium contained 10 g/L Bacto-tryptone (Difco Laboratories, Detroit, MI, United States), 5 g/L Bacto-yeast extract (Difco Laboratories), and 10 g/L NaCl. For plasmid maintenance throughout the initial incubation period, 50 mg/L of kanamycin and 50 mg/L of carbenicillin were added.

**FIGURE 1:**
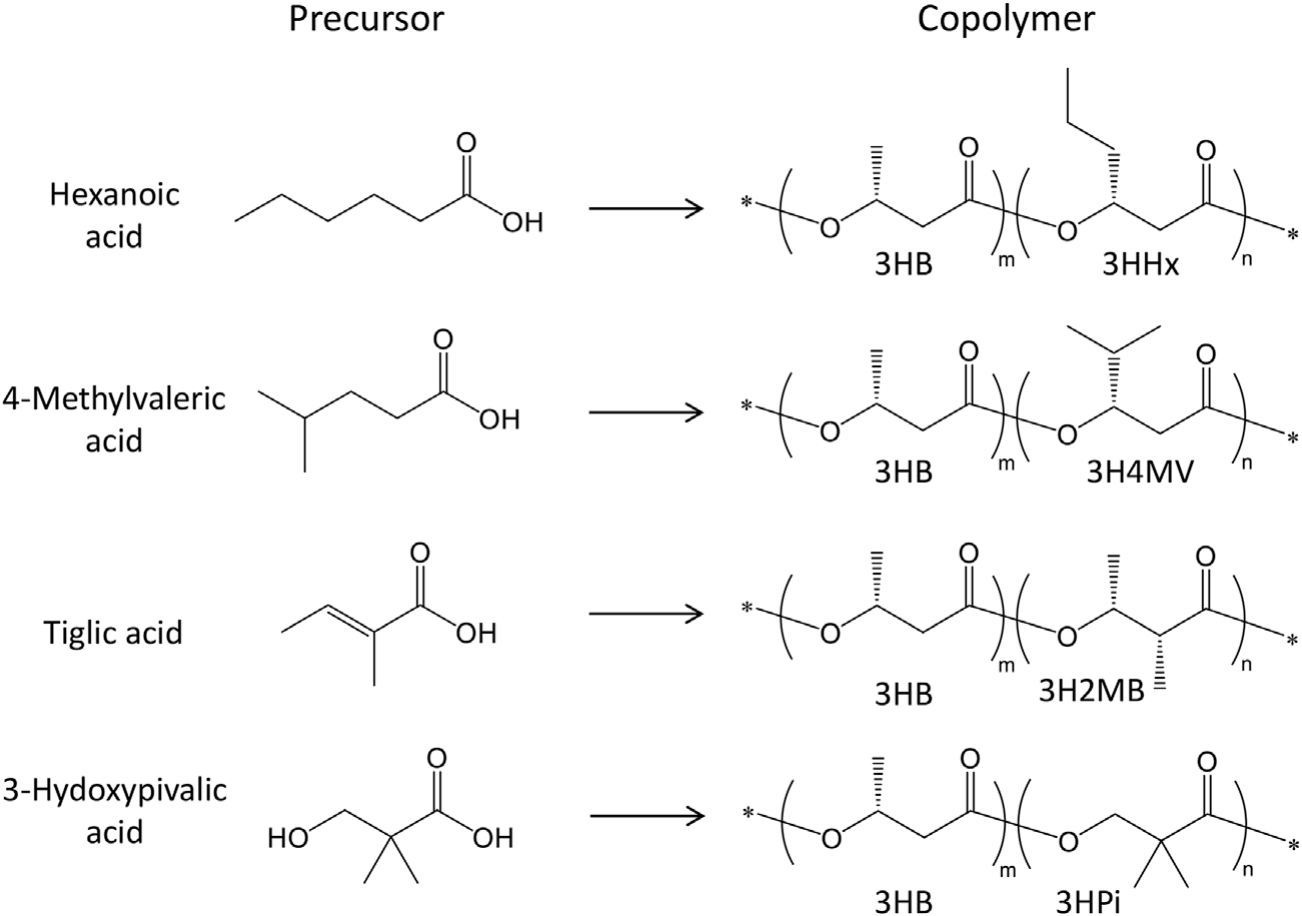
Chemical structure of PHA copolymers and precursors.

Inoculations for PHA production were started with 5 mL of seed culture added to 500 mL shake flasks containing 95 mL of modified M9 medium (Furutate et al., 2021) (final volume:100 mL and 5% inoculum). The modified M9 medium comprised of 17.1 g/L Na_2_HPO_4_·12H_2_O, 3 g/L KH_2_PO_4_, 0.5 g/L NaCl, 2 mL of 1 M MgSO_4_·7H_2_O, 0.1 mL of 1 M CaCl_2_, and 2.5 g/L Bacto-yeast extract. For plasmid maintenance during PHA production, 50 mg/L of kanamycin and 50 mg/L of carbenicillin were added. Additionally, 1 mM isopropyl-β-D-thiogalactopyranoside (IPTG) was used to induce *phaJ and pct* gene expression. The P(3HB) homopolymer was synthesized from 20 g/L glucose, which was added at the beginning of the culture at 30°C for 72 h. For the synthesis of 3HB-based copolymers, the total incubation time was set to 76 h, in which an initial step for 4 h at 30°C with reciprocal shaking (130 rpm) was performed before the addition of IPTG, precursors, and glucose, and further cultured for 72 h. Hexanoic acid, 4-methylvaleric acid, *trans*-2-methylbut-2-enoic acid (tiglic acid), and 2,2-dimethyl-3-hydroxypropionic acid (3-hydroxypivalic acid), which had previously been converted to their respective sodium salts, were used as precursors for the 3HHx, 3H4MV, 3H2MB, and 3HPi units, respectively (Füchtenbusch et al., 1998; Tanadchangsaeng et al., 2009; Watanabe et al., 2015). These precursors are known to inhibit cell growth, and a high concentration of glucose can repress *phaJ* and *pct* genes, otherwise induced by IPTG. Thus, lower concentrations of glucose and the precursors were added intermittently to the culture medium (at 4, 28, and 52 h). A total of 7.5 g/L glucose (2.5 g/L each time) and 0.6 g/L precursors (0.2 g/L each time) were added throughout the main incubation period. Finally, cells were harvested by centrifugation and lyophilized for further analysis. The relationship between the precursors used and biosynthesized polymers is shown in Figure 1.

### Site-directed mutagenesis

To construct mutated *phaC*_*Ps*_, a substitution (N175G) was introduced into the gene by overlap extension PCR (Supplementary Figure S2) (Warrens et al., 1997). The primers for amino acid substitution were designed and chemically synthesized as follows:5′-GGCGGCCGCTCTAGAACTAGTGGATCCCGGGGCAA-3′ and 5′- CAC​TAA​GTT​TTG​ACCGCCGTT​CTC​CAA​GGT-3′ for an amplification of the 1.4-kb fragment, 5′-GCG​CTT​GGA​GGC​CGG​CAC​CG-3′ and 5′- GTG​ACC​TTG​GAG​AACGGCGGT​CAA​AAC​TTA-3′ for an amplification of the 2.3-kb fragment. The underlined sequence in the primer indicates the codon used to replace Asn175 (AAT) with Gly (GGC). The resulting plasmid carrying the mutated gene was introduced into *E. coli* LSBJ along with pTTQ-PCT for PHA biosynthesis analysis.

### Analysis of PHA

The dry cell weight was gravimetrically measured after centrifuging the culture medium at 6,000 × *g* for 10 min at room temperature three times (once for collecting the cells, discarding the medium, and twice to wash away the remaining salts with water) and lyophilized for approximately 3 days.

PHA content, PHA yield, and 3HA monomer composition were determined by gas chromatography (GC) using a Shimadzu GC-2014s instrument (Shimadzu, Kyoto, Japan) with a flame ionization detector. Lyophilized cells were methanolyzed to convert PHA into 3HA-methyl ester constituents in the presence of 15% sulfuric acid for GC analysis. The methanolysis reaction was carried out at 100°C for 140 min, except for 3H2MB- and 3HPi-containing polymers, for which the reaction time was set to 8 h to increase the reaction yield. The methanolyzed samples were allowed to cool to room temperature, and 1 mL of deionized water was added to separate the polar components from the non-polar components. The non-polar fraction containing 3HA-methyl ester was filtered, and an equal volume of chloroform solution containing 0.1% (w/v) methyl-*n*-octanoate as an internal standard was added to prepare the final sample for GC analysis. The samples were injected through the GC capillary column InertCap 1 (30 m × 0.25 mm, GL Science, Tokyo, Japan). The column temperature was initially set at 90°C for 2 min, increased to 110°C at a rate of 5°C/min, and then increased to 280°C at a rate of 20°C/min. The signal peak areas obtained were calculated for the total PHA content and 3HA monomer composition.

The molecular weight of P(3HB) synthesized using various PhaC enzymes was determined by gel permeation chromatography (GPC) using a Shimadzu Nexera GPC system with an RI-504 refractive index detector (Shodex, Tokyo, Japan) equipped with two KF-406 LHQ joint-columns (at 40°C, Shodex, Tokyo, Japan). Chloroform was used as the mobile phase at a flow rate of 0.3 mL/min. The sample concentration and injection volume were set at 1 mg/mL and 10 μL, respectively. Polystyrene standards with low polydispersity were also analyzed as reference standards to construct a calibration curve.

## Results and discussion

### Identification of new PhaC enzymes using BLAST

A BLASTP search was performed against the protein sub-sections of the NCBI and DDBJ databases using the amino acid sequence of PhaC_Ac_, the first enzyme characterized by the natural copolymerization of 3HB and 3HHx monomers to PHA copolymers. Four PhaCs from the bacteria *Ferrimonas marina* (Katsuta et al., 2005), *Plesiomonas shigelloides* (Ferguson and Henderson 1947; Janda et al., 2016), *Shewanella pealeana* (Leonardo et al., 1999), and *Vibrio metschnikovii* (Lee et al., 1978) were selected for further evaluation, because of the diversity of the N-terminal region such as positions 149 and 171 in PhaC_Ac_. These PhaCs were identified as Class I PHA synthases, which have a high potential for synthesizing scl-mcl PHA copolymers in a manner similar to PhaC_Ac_, based on their homology. Although these bacteria were discovered long ago, their ability to produce PHA has not yet been studied.

**FIGURE 2:**
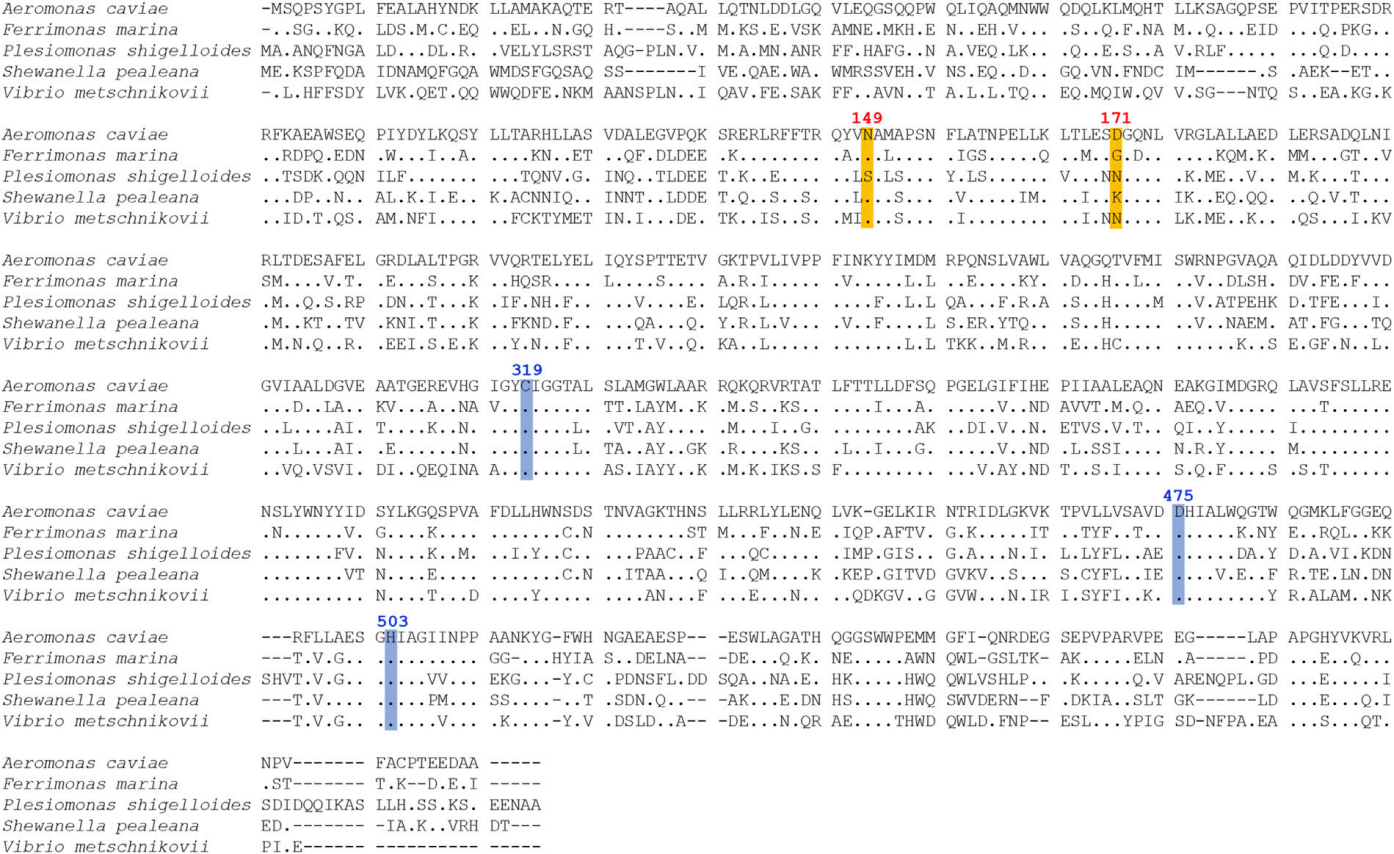
Multiple sequence alignment of PHA synthases (PhaCs) from *Aeromonas caviae*, *Ferrimonas marina, Plesiomonas shigelloides*, *Shewanella pealeana*, and *Vibrio metschnikovii*. The active site residues of PhaC_Ac_, cysteine (C^319^), aspartic acid (D^475^), and histidine (H^503^) are highlighted in blue. For the PhaC_Ac_NSDG variant, the positions of two amino acid substitutions (N^149^ replaced with S and D^171^ replaced with G) are highlighted in orange.

**FIGURE 3:**
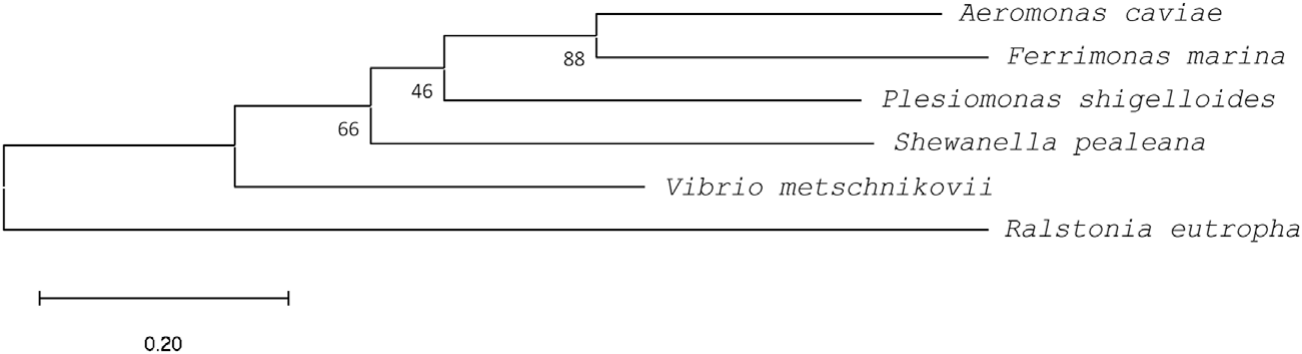
A phylogenetic tree of PhaCs rooted by outgroup (PhaC from *Ralstonia eutrophus*, WP_011615085). Sequences were aligned using ClustalW, and the phylogenetic tree was generated using MEGA11 software. PhaCs from *Aeromonas caviae* (BAA21815), *Ferrimonas marina* (WP_067661665)*, Plesiomonas shigelloides* (WP_116546999), *Shewanella pealeana* (WP_012154995), and *Vibrio metschnikovii* (WP_154168902) were used. Bootstrap values (expressed as percentages of 1,000 replications) are shown at the branch points. Scale bar = 0.2 substitution per amino acid position.

A comparison with the amino acid sequence of PhaC_Ac_ revealed that the four PhaC enzymes identified in this study shared 85%–91% similarity and approximately 55% identity with PhaC_Ac_ (Table 1). Multiple sequence alignment of PhaCs is shown in Figure 2. All new PhaCs have a PhaC box sequence at the active site, which is typically described as G-X-C-X-G-G (where X is an arbitrary amino acid), and cysteine (Cys^319^ in PhaC_Ac_) is the active center (Nambu et al., 2020). In PhaC_Ac_, the active sites Cys^319^, Asp^475^, and His^503^ have been proposed to form a catalytic triad (Tsuge et al., 2007a), which are all conserved in the newly identified PhaC enzymes. In contrast, PhaC from *P. shigelloides* has a primary sequence of approximately 30 amino acid residues greater than that of others and exhibits relatively low sequence homology in the C-terminal region. The phylogenetic tree shown in Figure 3 indicates that PhaC from *F. marina* is closely related to PhaC_Ac_, whereas PhaC enzymes from *S. pealeana* and *V. metchniskovii* are evolutionarily distinct. PhaC from *P. shigelloides* is neither closely related nor evolutionarily distant from PhaC_Ac_. To the best of our knowledge, no study has explored PhaC enzymes isolated from these bacteria for PHA production. Thus, genes encoding the four PhaC enzymes were chemically synthesized with optimized codon usage in *E. coli*. The DNA sequences are included in Supplementary Information.

**TABLE 1:** Four PhaCs characterized in this study.

PhaC from	Abbreviation	Accession	Protein size (amino acids)	Homology to PhaC_Ac_	
				Identity	Similarity
*Ferrimonas marina*	PhaC_Fm_	WP_067661665	592	58% (341/585)	91% (534/585)
*Plesiomonas shigelloides*	PhaC_Ps_	WP_116546999	623	54% (324/595)	86% (512/595)
*Shewanella pealeana*	PhaC_Sp_	WP_012154995	584	53% (303/564)	88% (499/564)
*Vibrio metschnikovii*	PhaC_Vm_	WP_154168902	590	52% (306/580)	85% (494/580)

PhaC_Ac_: PhaC from *Aeromonas caviae* (Accession BAA21815) with a protein size of 594 aa.

### P(3HB) synthesis in recombinant *E. coli* expressing PhaC enzymes

The biosynthesis of P(3HB) from 20 g/L glucose using one of the four PhaC enzymes is summarized in Table 2. P(3HB) accumulation ranging from 38.4 wt% to 54.2 wt% was achieved using the new PhaC enzymes, which was comparable to PhaC_Ac_ and its variant PhaC_Ac_NSDG. Thus, all newly identified PhaC enzymes showed great potential as biocatalysts for P(3HB) production. However, PhaCs from *P. shigelloides* (PhaC_Ps_) showed the highest P(3HB) accumulation among the wild-type PhaCs tested.

**TABLE 2:** Biosynthesis of P(3HB) from glucose by *E. coli* LSBJ expressing various PhaCs.

PhaC	Dry cell wt. (g/L)	P(3HB) content (wt%)	P(3HB) yield (g/L)	Molecular weight	
				*M*_*w*_ (×10^5^)	PDI
*A. caviae*	2.66 ± 0.02	39.8 ± 1.4	1.06 ± 0.04	8.5 ± 0.4	2.58 ± 0.36
*A. caviae*	3.83 ± 0.04	60.1 ± 4.3	2.30 ± 0.15	13.2 ± 0.5	2.45 ± 0.17
NSDG variant					
*F. marina*	2.77 ± 0.04	42.3 ± 1.1	1.17 ± 0.04	24.0 ± 3.0	1.39 ± 0.12
*P. shigelloides*	3.31 ± 0.02	54.2 ± 1.0	1.80 ± 0.03	34.4 ± 2.7	1.46 ± 0.13
*P. shigelloides*	3.59 ± 0.07	63.5 ± 1.4	2.28 ± 0.07	31.8 ± 5.9	1.37 ± 0.04
NG variant					
*S. pealeana*	2.39 ± 0.02	38.4 ± 5.0	0.92 ± 0.13	22.6 ± 1.6	1.54 ± 0.25
*V. metschnikovii*	3.08 ± 0.10	49.0 ± 2.2	1.51 ± 0.12	19.7 ± 1.5	2.62 ± 0.10

*E. coli* LSBJ harboring pBBR1-phaCsAB_Re_J_Ac_ was incubated in the modified M9 medium containing 20 g/L glucose as a carbon source. The values of dry cell weight, PHA content, and molecular weight were the averages of three independent experiments. P(3HB): poly(3-hydroxybutyrate). The NSDG variant of *A. caviae* PhaC had a double mutation of N149S and D171G. The NG variant of *P. shigelloides* PhaC had a single mutation of N175G. PDI is polydispersity index (*M*_*w*_/*M*_*n*_).

The molecular weight is a crucial aspect in determining the suitability of a material for various commercial uses (Sudesh et al., 2000). The weight-average molecular weight (*M*_*w*_) is more closely related to material properties than the number-average molecular weight (*M*_*n*_). For PHA, ultrahigh molecular weight polymers can form strong fibers (Tsuge, 2016), thus meeting the requirements for practical use. In addition, a low polydispersity index (PDI) (Tsuge, 2016) also plays a significant role in determining the suitability of PHA for specific applications. However, not all PhaC enzymes can synthesize PHAs with high *M*_*w*_ and low PDI. In this study, PhaC_Ps_ synthesized P(3HB) with an ultrahigh *M*_*w*_, which exceeded 3 × 10^6^, with a relatively low PDI below 1.5 (Table 2). Moreover, the other two identified PhaC enzymes from *F. marina* and *S. pealeana* could also synthesize P(3HB) with *M*_*w*_ of approximately 2 × 10^6^ with PDIs ranging from 1.3 to 1.5. PhaC_Vm_ from *V. metschnikovii* proved to be an exception, with PDI >2.5. The currently available PhaC_Ac_ is highly sensitive to ethanol (Hiroe et al., 2015), which is a metabolite of some bacteria, including *E. coli,* and functions as a chain transfer agent to terminate polymerization reactions (Tsuge, 2016), resulting in the synthesis of relatively low-molecular-weight PHA. The new PhaCs reported in this study may be less sensitive toward ethanol, thereby producing PHA with high *M*_*w*_ and low PDI. These new PhaC enzymes, especially PhaC_Ps_, exhibited superior *M*_*w*_ and PDI values compared with PhaC_Ac_ and its NSDG variant, which could benefit PHA processing and material properties.

### PHA copolymer synthesis by recombinant *E. coli* expressing PhaC enzymes

The new PhaC enzymes were evaluated for their substrate specificities alongside PhaC_Ac_ and its NSDG variant for incorporating 3HHx, 3H4MV, 3H2MB, and 3HPi monomers. Biosynthesis was performed using four precursors (hexanoic acid, 4-methylvaleric acid, tiglic acid, and 3-hydroxypivalic acid) in the presence of glucose (Figure 1). These precursors are toxic to cells, thus inhibiting cell growth and subsequently lowering PHA accumulation in bacteria. As PHA production is associated with cell growth (Sudesh et al., 2000), it is imperative to eliminate or reduce the risk of toxicity induced by such precursors. Therefore, the precursors were introduced into the culture medium after 4 h, once substantial cell growth was achieved, mainly for better tolerance (Furutate et al., 2021). Meanwhile, a high glucose concentration can cause catabolic repression of *phaJ* and *pct* genes induced by IPTG (Furutate et al., 2021); thus, the glucose concentration was maintained at a minimum to promote cell growth only. Glucose and its precursors were intermittently added to allow for better uptake of the second monomer, with no or fewer unanticipated effects on the cells. The details of the biosynthesis results are summarized in Tables 3–6.

**TABLE 3:** Biosynthesis of P (3HB-*co*-3HHx) by *E. coli* LSBJ expressing various PhaCs from glucose and hexanoic acid.

PhaC	Dry cell wt. (g/L)	PHA content (wt%)	PHA yield (g/L)	PHA composition (mol%)	
				3HB	3HHx
*A. caviae*	1.93 ± 0.04	16.9 ± 0.8	0.30 ± 0.01	86.7 ± 0.8	13.3 ± 0.8
*A. caviae*	2.12 ± 0.02	25.2 ± 1.3	0.51 ± 0.03	78.2 ± 1.4	21.8 ± 1.4
NSDG variant					
*F. marina*	1.92 ± 0.03	23.6 ± 1.2	0.45 ± 0.02	90.5 ± 1.0	9.5 ± 1.0
*P. shigelloides*	1.78 ± 0.04	19.1 ± 0.3	0.34 ± 0.01	89.1 ± 1.2	10.9 ± 1.2
*P. shigelloides*	1.80 ± 0.05	11.9 ± 0.7	0.21 ± 0.12	90.0 ± 0.2	10.0 ± 0.2
NG variant					
*S. pealeana*	1.68 ± 0.06	12.2 ± 0.5	0.20 ± 0.01	89.5 ± 0.5	10.5 ± 0.5
*V. metschnikovii*	1.82 ± 0.01	18.7 ± 0.9	0.34 ± 0.02	96.0 ± 0.2	4.0 ± 0.2

*E. coli* LSBJ harboring pBBR1-phaCsAB_Re_J_Ac_ and pTTQ-PCT was incubated in the modified M9 containing 7.5 g/L glucose (2.5 g/L × 3 times) and 0.6 g/L hexanoic acid (0.2 g/L × 3 times), which were added at 4, 28, and 52 h. The values of dry cell weight, PHA content, and PHA composition were the averages of three independent experiments. The NSDG variant of *A. caviae* PhaC had a double mutation of N149S and D171G. The NG variant of *P. shigelloides* PhaC had a single mutation of N175G. 3HB: 3-hydroxybutyrate; 3HHx: 3-hydroxyhexanoate.

**TABLE 4:** Biosynthesis of P (3HB-*co*-3H4MV) by *E. coli* LSBJ expressing various PhaCs from glucose and 4-methylvaleric acid.

PhaC	Dry cell wt. (g/L)	PHA content (wt%)	PHA yield (g/L)	PHA composition (mol%)	
				3HB	3H4MV
*A. caviae*	1.66 ± 0.04	17.8 ± 1.2	0.30 ± 0.03	95.4 ± 0.3	4.6 ± 0.3
*A. caviae*	1.68 ± 0.01	18.0 ± 1.4	0.30 ± 0.02	93.7 ± 0.5	6.3 ± 0.5
NSDG variant					
*F. marina*	1.92 ± 0.02	27.0 ± 2.9	0.52 ± 0.06	98.5 ± 0.1	1.5 ± 0.1
*P. shigelloides*	1.78 ± 0.03	20.9 ± 0.8	0.37 ± 0.01	97.5 ± 0.2	2.5 ± 0.2
*P. shigelloides*	1.86 ± 0.01	16.6 ± 4.9	0.31 ± 0.10	96.3 ± 0.1	3.7 ± 0.1
NG variant					
*S. pealeana*	1.69 ± 0.01	18.4 ± 1.2	0.31 ± 0.02	100	ND
*V. metschnikovii*	1.85 ± 0.02	20.7 ± 2.1	0.38 ± 0.04	98.1 ± 0.4	1.9 ± 0.4

*E. coli* LSBJ harboring pBBR1-phaCsAB_Re_J_Ac_ and pTTQ-PCT was incubated in the modified M9 containing 7.5 g/L glucose (2.5 g/L × 3 times) and 0.6 g/L 4-methylvaleric acid (0.2 g/L × 3 times), which were added at 4, 28, and 52 h. The values of dry cell weight, PHA content, and PHA composition were the averages of three independent experiments. The NSDG variant of *A. caviae* PhaC had a double mutation of N149S and D171G. The NG variant of *P. shigelloides* PhaC had a single mutation of N175G. 3HB: 3-hydroxybutyrate; 3H4MV: 3-hydroxy-4-methylvalerate.

**TABLE 5:** Biosynthesis of P (3HB-*co*-3H2MB) by *E. coli* LSBJ expressing various PhaCs from glucose and tiglic acid.

PhaC	Dry cell wt. (g/L)	PHA cont. (wt%)	PHA yield (g/L)	PHA composition (mol%)	
				3HB	3H2MB
*A. caviae*	2.02 ± 0.04	23.1 ± 0.6	0.47 ± 0.02	95.7 ± 0.1	4.3 ± 0.1
*A. caviae*	2.33 ± 0.03	29.5 ± 2.6	0.69 ± 0.06	95.1 ± 0.2	4.9 ± 0.2
NSDG variant					
*F. marina*	2.30 ± 0.08	31.9 ± 1.2	0.74 ± 0.05	99.3 ± 0.0	0.7 ± 0.0
*P. shigelloides*	2.21 ± 0.08	29.5 ± 2.6	0.76 ± 0.05	97.9 ± 0.2	2.1 ± 0.2
*P. shigelloides*	2.24 ± 0.03	30.0 ± 1.2	0.67 ± 0.03	94.6 ± 0.1	5.4 ± 0.1
NG variant					
*S. pealeana*	1.88 ± 0.06	20.3 ± 0.3	0.38 ± 0.01	100	ND
*V. metschnikovii*	2.31 ± 0.03	30.4 ± 2.7	0.72 ± 0.01	99.5 ± 0.0	0.5 ± 0.0

*E. coli* LSBJ harboring pBBR1-phaCsAB_Re_J_Ac_ and pTTQ-PCT was cultured in the modified M9 medium containing 7.5 g/L glucose (2.5 g/L × 3 times) and 0.6 g/L tiglic acid (0.2 g/L × 3 times), which were added at 4, 28, and 52 h. The values of dry cell weight, PHA content, and PHA composition were the averages of three independent experiments. The NSDG variant of *A. caviae* PhaC had a double mutation of N149S and D171G. The NG variant of *P. shigelloides* PhaC had a single mutation of N175G. 3HB: 3-hydroxybutyrate; 3H2MB: 3-hydroxy-2-methylbutyrate.

**TABLE 6:** Biosynthesis of P (3HB-*co*-3HPi) by *E. coli* LSBJ expressing various PhaCs from glucose and 3-hydoxypivalic acid.

PhaC	Dry cell wt. (g/L)	PHA cont. (wt%)	PHA yield (g/L)	PHA composition (mol%)	
				3HB	3HPi
*A. caviae*	2.01 ± 0.03	16.3 ± 1.4	0.33 ± 0.03	94.2 ± 0.6	5.8 ± 0.6
*A. caviae*	2.07 ± 005	19.4 ± 1.2	0.40 ± 0.03	79.9 ± 1.1	20.1 ± 1.1
NSDG variant					
*F. marina*	2.22 ± 0.04	23.1 ± 0.3	0.51 ± 0.01	97.5 ± 0.6	2.5 ± 0.6
*P. shigelloides*	1.98 ± 0.05	16.7 ± 1.3	0.33 ± 0.03	89.8 ± 0.4	10.1 ± 0.4
*P. shigelloides*	1.91 ± 0.22	15.0 ± 2.7	0.29 ± 0.06	88.4 ± 0.5	11.6 ± 0.5
NG variant					
*S. pealeana*	1.80 ± 0.08	9.7 ± 0.1	0.17 ± 0.01	97.9 ± 0.4	2.1 ± 0.4
*V. metschnikovii*	2.15 ± 0.03	24.2 ± 0.9	0.52 ± 0.02	97.2 ± 0.2	2.8 ± 0.2

*E. coli* LSBJ harboring pBBR1-phaCsAB_Re_J_Ac_ and pTTQ-PCT was incubated in the modified M9 medium containing 7.5 g/L glucose (2.5 g/L × 3 times) + 0.6 g/L 3-hydroxypivalic acid (0.2 g/L × 3 times), which were added at 4, 28, and 52 h. The values of dry cell weight, PHA content, and PHA composition were the averages of three independent experiments. The NSDG variant of *A. caviae* PhaC had a double mutation of N149S and D171G. The NG variant of *P. shigelloides* PhaC had a single mutation of N175G. 3HB: 3-hydroxybutyrate; 3HPi: 3-hydroxypivalate.

All PhaCs, except PhaC from *S. pealeana* (PhaC_Sp_), were able to incorporate all targeted monomers (3HHx, 3H4MV, 3H2MB, and 3HPi). PhaC_Sp_ copolymerized 3HB with 3HHx or 3HPi, but not 3H4MV or 3H2MB. The number of PhaC enzymes with broad substrate specificity is scarce; thus, the new PhaC enzymes reported in this study are highly intriguing for future studies. Furthermore, PHAs containing *α*-carbon methylated units are potentially attractive bio-based materials (Füchtenbusch et al., 1998; Furutate et al., 2021); thus, PhaCs with the ability to polymerize 3H2MB and 3HPi are of great interest. PhaC_Ps_ demonstrated superior performance in the polymerization of 3HPi to PhaC_Ac_. Additionally, all PhaC_Ps_-expressing strains showed higher PHA content than PhaC_Ac_-expressing strains. Therefore, the potential of PhaC_Ps_ was further explored using site-directed mutagenesis.

### Generation and evaluation of PhaC_Ps_NG variant

*In vitro* evolution of PhaC is a powerful approach for enhancing the productivity and quality of PHA (Kichise et al., 2002; Taguchi and Doi, 2004). For instance, PhaC_Ac_NSDG, a variant of PhaC_Ac_, exhibits enhanced performance (such as production yield and substrate specificity) compared to that of the wild-type enzyme (Tsuge et al., 2007b). In addition, various studies have proven the efficacy of PhaC engineering in PHA production towards the formation of super biocatalysts for tailor-made PHAs (Taguchi and Doi, 2004; Nomura and Taguchi, 2007). Therefore, PhaC_Ps_, which exhibited the best performance among the new PhaCs, were selected for site-directed mutagenesis to study their potential positive effects on PHA production. Considering that the double mutation of PhaC_Ac_NSDG, amino acid substitutions of N149S and D171G drastically enhanced the performance of the enzyme (Tsuge et al., 2007b; Harada et al., 2021), similar efforts were adopted to generate a PhaC_Ps_ variant. According to the alignment (Figure 2), PhaC_Ps_ naturally contain a serine residue at the corresponding position of 149 in PhaC_Ac_NSDG. Thus, a single amino acid substitution was performed in PhaC_Ps_ in which asparagine 175 was changed to glycine (N175G). The resultant variant was termed PhaC_Ps_NG, and its PHA production ability was examined.

Interestingly, PhaC_Ps_NG showed enhanced P(3HB) synthesis, while maintaining a high molecular weight (Table 2). PhaC_Ps_NG exhibited enhanced activity for the incorporation of the *α*-methylated monomer 3H2MB compared with the parent enzyme and PhaC_Ac_NSDG (Table 5). This indicates the potential of PhaC_Ps_NG to surpass the currently best-performing enzyme (PhaC_Ac_NSDG) for the incorporation of the 3H2MB monomer. Moreover, PhaC_Ps_NG was shown to have a better ability to incorporate 3H4MV and 3HPi than the parent enzyme but less so than PhaC_Ac_NSDG (Table 4 and Table 6). Finally, PhaC_Ps_NG exhibited an almost similar level of 3HHx incorporation as the parent enzyme, which was inferior to PhaC_Ac_NSDG (Table 3).

## Conclusion

In conclusion, four Class I PhaC enzymes from different bacteria were identified using BLASTP and were characterized for PHA production. To the best of our knowledge, this is the first report to characterize PhaC enzymes from *F. marina*, *P. shigelloides*, *S. pealeana*, and *V. metschnikovii*. These PhaCs exhibited a relatively high potential for polymerizing P(3HB) in recombinant *E. coli*. PhaC enzymes identified in this study, with the exception of PhaC_Sp_ from *S. pealeana,* were able to incorporate all the targeted monomers, namely 3HHx, 3H4MV, *α*-carbon methylated 3H2MB, and *α*-carbon dimethylated 3HPi. Among the four new PhaCs, PhaC_Ps_ from *P. shigelloides* displayed the best performance; thus, we attempted to further improve their attributes through protein engineering. The resultant variant PhaC_Ps_NG exhibited superior capability in polymerizing the 3H2MB monomer compared to PhaC_Ac_ and its NSDG variant. Furthermore, PhaC_Ps_NG showed the enhanced synthesis of P(3HB) with ultrahigh molecular weight and low PDI. Finally, these newly identified PhaC enzymes show great versatility, suggesting their potential as workhorse enzymes for the industrial-scale production of 3HB-based copolymers.

## Data Availability

The datasets presented in this study can be found in online repositories. The names of the repository/repositories and accession number(s) can be found in the article/Supplementary Material.
