# Supplementary material for: Exploring Class I polyhydroxyalkanoate synthases with broad substrate specificity for polymerization of structurally diverse monomer units
Source: Front Bioeng Biotechnol. 2023 Feb 21;11:1114946. doi: 10.3389/fbioe.2023.1114946 (PMC9989198; doi:10.3389/fbioe.2023.1114946)
Supplement: Supplementary file 1 [file DataSheet1.pdf]

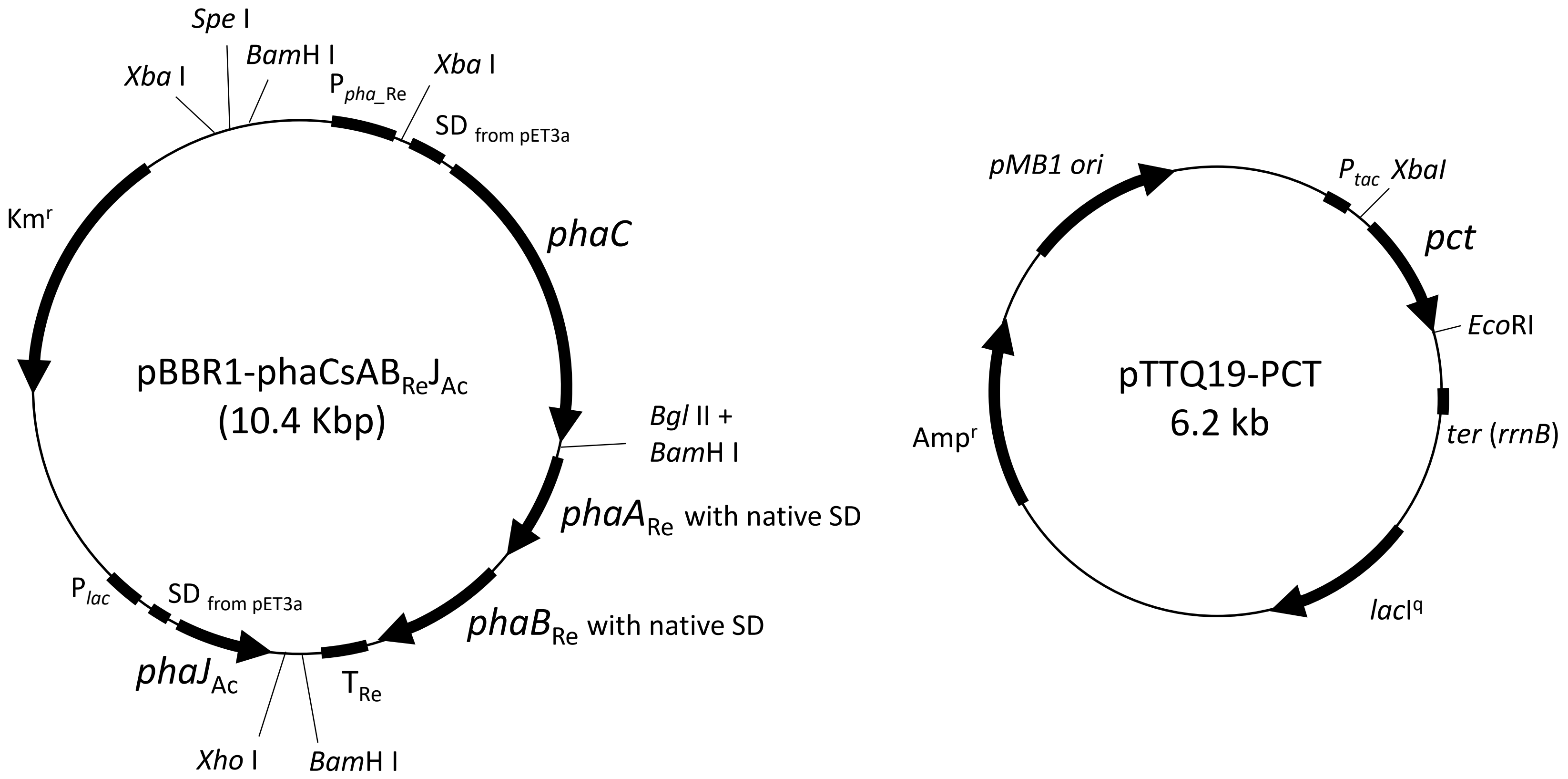

Figure S1: Schematic diagram of recombinant plasmid pBBR1-phaCsAB$_{Re}$J$_{Ac}$ used for the evaluation of PhaCs for their broad specificities and pTTQ19-PCT (Furutate et al., *J Polym Res*, 24, 221, 2017).

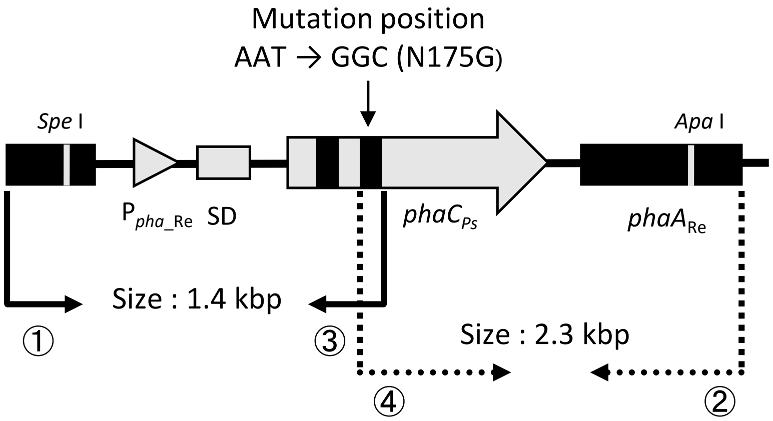

Figure S2: Schematic diagram depicting the insertion of point mutation using overlap extension PCR

Primer sequences

①	5'-GGCGGCCGCTCTAGAACTAGTGGATCCCGGGGCAA-3'
②	5'-GCGCTTGGAGGCCGGCACCG-3'
③	5'-CACTAAGTTTTGACC<u>GCC</u>GTTCTCCAAGGT-3'
④	5'-GTGACCTTGGAGAAC<u>GGC</u>GGTCAAAACTTA-3'

The underlined sequence in the primer indicates the codon used to replace Asn175 (AAT) with Gly (GGC).

PhaC from *Ferrimonas marina*

Chemically synthesized DNA sequence:

ATG AGC TCA GGC TCG TAC AAG CAG TTA CTG GAT AGC CTC ATG CAC TGT AAC GAG CAG TTG CTG GAA CTG GCG AAG AAC CAG GGG CAG CAT ACC TCG CAG GCG ATG ATG CAG AAG AGC CTG GAG GAT GTG TCG AAA GCG ATG AAC GAA GGC ATG AAA CAT CCG GAA AAC CTT ATC GAA CAC CAA GTG AAC TGG TGG CAG TCA CAA TTA CAG TTA TTT CAA AAC GCG ATG CTG AAA CAA GCG GGT CAG GAA ATT GAT CCC GTA ATT CAG CCG CCG AAA GGT GAT CGC CGC TTT CGT GAC CCG CAG TGG GAA GAT AAT CCG TGG TAC GAT TAC ATC AAA CAA GCG TAT CTG CTG ACG GCC AAG AAC TTA CTG GAA ACC GTT GAC CAA TTT GAG GAC CTG GAT GAA GAG TCA AAA GAA CGG CTC CGT TTC TTT ACC CGT CAA GCC GTG AAT GCT CTC GCC CCA AGC AAC TTC ATT GGT TCC AAT CCG GAA CTG CTT CAG TTA ACC ATG GAA TCT GGA GGC GAC AAC CTG GTT CGC GGA CTT AAA CAG ATG GCA AAA GAC ATG ATG CGC TCT GCC GGC ACC CTG AAT GTT AGC ATG ACC GAT GAA TCC GTG TTT ACC CTT GGA GAG GAT CTG GCA TCG ACT CCA GGG AAA GTG GTT CAC CAG AGC CGT CTG TAT GAG CTG CTT CAA TAT TCC CCG TCT ACC GAA ACG GTG GCG AAG CGC CCC ATT CTG ATT GTA CCG CCG TTT GTG AAC AAA TAC TAC ATT TTG GAT CTG CGC CCG GAG AAC TCA CTG GTT AAA TAC CTG GTT GAC CAA GGC CAT ACG GTG CTC ATG ATT AGC TGG GTA AAC CCG GAC CTC TCT CAT GCT GAC GTA GAT TTC GAA GAT TTC GTT GTC GAT GGT GTC ATC GAT GCA TTA CTG GCG GTG GAA AAG GTA ACG GGC GAA GCC GAA GTC AAT GCA GTT GGT TAT TGC ATT GGT GGC ACA GCT CTG ACC ACC GCT CTG GCA TAT ATG GCC GCG AAA CGC ATG AAA TCC CGC GTT AAA AGT GCC ACG CTG TTC ACT ACC ATT CTG GAC TTT GCG CAG CCA GGT GAA TTA GGG GTG TTT ATC AAC GAT GCC GTG GTC ACG GCT ATG GAA CAG CAG AAT GCG GAA CAA GGC GTG ATG GAC GGC CGC CAG TTG GCG GTC ACA TTC AGT TTA CTG CGT GAA AAT AAC TTG TAT TGG AAT TAC TAT GTG GAT GGG TAC CTG AAA GGC AAA AGT CCG GTG GCC TTC GAC CTG TTG CAC TGG AAT TGC GAT AAT ACT AAC GTC GCA GGC AAA ACT CAT AGC ACC ATG CTT CGC CGC TTC TAT TTG AAT AAC GAG CTC ATC CAA CCT GGT GCG TTT ACG GTC CGT GGC ACT AAA ATC GAT CTG GGT AAG ATC ACA ACG CCC ACT TAT TTT GTC AGT ACC GTG GAT GAC CAC ATT GCC CTG TGG AAA GGT AAC TAT GAA GGC ATG CGT CAG CTG GGC GGT AAG AAA ACA TTT GTT CTG GGA GAG TCG GGC CAT ATT GCC GGG ATT ATT AAT CCG CCT GGT GGA AAA TAT GGT CAT TAC ATC GCT AGT GGG GCA GAC GAA TTG AAT GCG GAT GAA TGG CTT GCG CAG GCC AAA CAC AAT GAG GGC TCC TGG TGG CCA GCA TGG AAT CAG TGG TTG GGC AGC CTG ACC AAA GCT AAA CCT GTC CCA GCA CGT GAA CTG AAC GAG GCA CTG CCT GAT GCG CCG GGT GAA TAT GTA CAG GTT CGG CTC AAC TCT ACG ACC GCC AAA GAT GAA GAG GCG ATC TGA

PhaC from *Plesiomonas shigelloides*

Chemically synthesized DNA sequence:

ATG GCA AGT GCG AAT CAG TTT AAC GGC GCG CTG GAC GCT CTG GCC GAT CTC AAT CGG AAA CTG GTC GAA CTG TAC CTC TCC CGC TCA ACG GCC CAA GGC
CCG CTG AAT CAG GTT CTC ATG CAG GCC AAT ATG AAC GAT GCA AAT CGC TTC TTC GAA CAT GCG TTC GGT CAA CCA AAT GCC TTA GTT GAA CAG CAG CTG
AAA TGG TGG CAG CAA CAG CTG GAA CTG TCC CAG CAC GCA GTG CTG CGC CTG TTT GGC CAG CCT AGT GAG CCG GTG ATC CAG CCC GAT CGT TCT GAT CGT
CGC TTT ACT TCT GAC AAG TGG CAG CAG AAT ATC CTG TTC GAT TAC CTG AAG CAG TCC TAT CTT CTG ACG ACA CAA AAT GTC TTA GGT TCC ATT AAC CAG
CTG GAA ACG CTG GAC GAG GAA ACC CGT AAG CGC CTG GAG TTC TTC ACG CGT CAG TAT CTG AGC GCG TTA TCG CCG TCG AAC TAT CTG CTG TCA AAC CCG
GAA TTG CTG AAA GTG ACC TTG GAG AAC **AAT** GGT CAA AAC TTA GTG AAA GGC ATG GAA CTG CTT GTC GAA GAT ATG GAG AAG TCA GCG GAC ACC CTG
AAT ATT CGC ATG ACA GAT CAG TCG AGC TTT CGT CCT GGT GAC AAC CTG GCG ACC ACA CCC GGT AAA GTA ATC TTT CGC AAT CAT CTG TTC GAG TTA ATT
CAG TAC GTC CCG ACT ACC GAA GAA GTC TTA CAA CGC CCT CTG TTG ATT GTG CCA CCG TTC ATC AAC AAA TTT TAC ATC CTT GAT TTA CAA GCG CAA AAT
AGC TTT GTT CGG TGG GCC GTA AGC CAG GGT CAT ACG GTC TTT ATG ATG AGC TGG GTG AAC GCC ACC CCG GAA CAC AAA GAC ATC ACC TTC GAA GAT TAC
GTG ATT GAC GGT GTA TTA GCT GCA CTT GAT GCC ATC GAA ACG GCG ACC GGG GAG AAA GAG GTG AAT GGC ATC GGC TAT TGC ATT GGA GGC ACC CTT CTT
TCG GTC ACC ATG GCG TAT CTG GCG GCA CGT CGC ATG AAA CAA CGT ATT CGT ACT GGC ACT CTG TTC ACG ACC TTA CTC GAT TTT GCC AAA CCG GGT GAC
ATT GGC GTG TTT ATC AAC GAA GAA ACC GTG TCA GCT GTT GAG ACT CAG AAT CAA ATC AAA GGG TAT ATG GAC GGT CGC CAA ATT GCG GTG AGC TTC AGC
CTT CTC CGC GAA AAC TCG CTG TAC TGG AAT TAT TTT GTG GAT AAC TAT CTG AAG GGC AAA TCT CCA ATG GCG TTT GAC ATT CTG TAT TGG AAC TGT GAT
TCC ACC AAC GTT CCC GCA GCC TGC CAT AAC TTT CTC TTG CGT CAG TGC TAT TTG GAA AAC CAG CTG ATT ATG CCG GGC GGC ATT TCA ATC CGC GGA ACC
GCG ATC GAT TTG AAC AAA ATC AAA CTG CCG TTG TAT TTC CTG AGT GCC GCC GAA GAT CAC ATT GCT TTG TGG GAT GCA ACG TAC GAT GGC GCG AAA GTC
ATT GGA AAA GAC AAT TCC CAT GTT ACA TTT GTT CTC GGT GAA AGC GGC CAT ATT GCG GGT GTT GTA AAT CCA CCG GAG AAA GGG AAA TAC GGC TAT TGG
TGT AAT CCC GAT AAC AGC TTT CTG CCG GAT GAT TCT CAA GCT TGG CTG AAC GCC GCT GAA CAC CAC AAA GGA AGC TGG TGG CCT CAT TGG CAG CAA TGG
CTG GTG AGT CAT CTG CCG GAA GGT TCT AAA CCG GTA CCA GCT CGT CAG CCG GTT GCA CGC GAA AAC CAA CCG TTG CTC GGG GAC GCG CCT GGG GAA TAC
GTG AAA GTA CGC ATT TCG GAT ATT GAC CAG CAG ATT AAG GCA AGC CTG CTG CAC CCA AGT TCT GAA AAG AGT GCC GAG GAA AAC GCG GCA TGA

$PhaC_{Ps}$ NG variant: the position for amino acid substitution is underlined and shown in red, AAT → GGC (N175G)

PhaC from *Shewanella pealeana*

Chemically synthesized DNA sequence:

ATG GAG AGC AAA AGC CCG TTT CAG GAC GCC ATT GAT AAT GCG ATG CAA TTC GGT CAA GCA TGG ATG GAC TCC TTT GGC CAG TCT GCG CAG TCA TCC
ATC GTT GAG ACT CAG GCC GAA GAT TGG GCC CAG TGG ATG CGT TCC AGT GTT GAG CAT CCA GTG AAC TCT ATC GAA CAA CAA ATG GAT TGG TGG GGT
CAG CAA GTG AAC CTG TTT AAC GAC TGC ATT ATG TCG AGC CCG GCG GAG AAA GAG ACA GAT CGG CGC TTT AAA GAT CCA GCG TGG AAC GAA CAA
GCG CTG TAC AAG TAT ATT AAG GAA TCG TAC AAA CTG GCG TGT AAT AAC ATT CAG GCA AGC ATT AAC AAT ACT GAA GGC CTT GAT GAT GAA ACC
CGT CAA CGT CTG TCG TTC TTT AGC CGT CAG TAC CTG AAT GCC ATG TCA CCG AGC AAC TTT GTG GCA ACT AAC CCG GAA ATT ATG AAA CTG ACC ATC
GAA TCG AAA GGT CAA AAC TTG ATC AAA GGG CTG GAA CAG CTG CAA CAG GAC CTT GAG CAA TCT GTG GAC ACC TTG AAT ATC CGC ATG ACC GAT
AAA ACC GCT TTT ACC GTG GGC AAG AAC ATC GCT ACG ACG CCT GGC AAA GTT GTC TTT AAG AAC GAT CTG TTT GAA CTG ATT CAG TAT CAG GCC ACG
ACC GAA CAG GTG TAC AAG CGG CCG TTG TTA GTG GTA CCG CCT TTT GTC AAC AAA TTC TAC ATT ATG GAT CTC AGC CCT GAA CGC AGC TAT ACG CAG
TGG TTA GTC AGC CAG GGT CAT ACC GTA TTT ATG ATT TCT TGG GTG AAT CCG AAT GCA GAG ATG GCG GCA ACG GAT TTC GGT GAC TAT GTC ACG CAG
GGC GTA ATC CTG GCC TTA GAC GCG ATT GAA GCA GAA ACG GGC GAA CGC GAA GTT AAT GGC ATT GGG TAT TGC ATT GGT GGG ACC CTT CTG ACG
GCG GCC ATG GCG TAC TTA GCC GGA AAA CGT CGC AAA CAG CGT GTC AAA TCT GCG ACT TTG CTG ACC ACA ATC CTG GAT TTC GGC CAA CCG GGT GAA
CTC GGG GTG TTT ATC AAC GAT CCG CTG ATC AGT AGT ATT GAA GCG CAG AAT AAC GCA CGT GGA TAT ATG GAT GGT CGC CAA ATG GCA GTA TCA TTC
AGC CTC TTA CGC GAG AAT AGT CTG TAT TGG AAC TAT TAT GTG ACC AAC TAC CTC AAA GGC GAA TCT CCC GTT GCC TTC GAC TTG TTG CAC TGG AAT
TGT GAC AAT ACA AAC ATC ACT GCC GCG ACC CAT AAC CAG ATT CTG CGC CAG ATG TAT CTG GAG AAT AAG CTC AAA GAA CCG GGA GGG ATT ACC
GTT GAT GGT GTG AAA GTT GAC CTG AGT AAA GTC AAG AGC CCG TGC TAT TTT CTG TCG GCC ATT GAG GAC CAC ATC GCA GTT TGG GAG GGC ACA TTC
CGC GGC ACT GAA CTG CTG AAC GGC GAT AAC ACC TTC GTA TTA GCG GAA TCC GGT CAC ATT GCC GGT CCC ATG AAT CCG CCG AGT TCG AAC AAA TAC
GGA TTT TGG ACC AAT TCC GAT AAT GCT CAG TCA CCC GCG AAA TGG CTG GCT GAG GCG GAT AAT CAC TCC GGT TCA TGG TGG CCA CAT TGG CAG TCG
TGG GTT GAC GAA CGC AAT TTC AGT GAT AAA ATC GCT GCA CGC AGC CTG ACG GGC AAA CTT GAC GCT CCA GGC GAA TAC GTG AAA CAG CGC ATT
GAA GAT GTG ATT GCT CCT AAA GAA GAA GTC CGT CAT GAT ACA TGA

PhaC from *Vibrio metschnikovii*

Chemically synthesized DNA sequence:

ATGCTCCAACACTTCTTCAGTGATTACCTGGTGAAACTGCAGGAAACTAACCAGCAGTGGTGGCAGGATTTTGAGGCCAATAAGATGGCCGCGAATAGCCCGCTGAAT
CAGGCAATTCAAGCGGTTAACTTTGAAGATTCTGCGAAATTCTTTGAGCAGGCAGTAAATCAGCCAACAGCCCTACTGCAACTTCAAACCCAGTGGTGGGAACAACAG
ATGCAGATCTGGCAGCAAGTGGTCTTAAGCGGCAACACCCAAAGCGTTATCGAAGCGGAAAAAGGCGATAAACGCTTTATCGATGAAACGTGGCAGAGTCAAGCGAT
GTATAACTTCATTAAACAGTCCTATCTGCTCTTCTGCAAAACCTACATGGAGACTATCAATGCAATCGAAGGAGTGGATGAAAAAACCAAAGAGCGTATCTCGTTCTTT
AGTCGGCAGATGATCAATGCGATGTCTCCGTCTAATTTCATTGCTACGAACCCTGAACTGTTGAAGCTGACCATAGAGAACAACGGTCAAAACTTACTGAAAGGCATG
GAGCTGCTGAAAGAAGATTTGCAGTCAAGCGCGGATATACTGAAAGTGCGTATGACGAACGAACAGGCCTTTCGCTTGGGCGAAGAGATCGCTTCTACTGAGGGCAAA
GTGGTGTATCGGAATGAACTTTTCGAACTGATCCAGTATACCCCTGTTACCGAACAGGTTAAAGCCACTCCGCTGCTAATTGTACCCCCGTTTATCAACAAGTACTATAT
CCTGGATCTCACGAAAAAGAACAGTATGGTGCGCTGGCTGGTTGAGCAGGGTCATTGTGTCTTCATGATCTCTTGGAGAAATCCAGGTAAAGCCCAAAGCGAGATTGG
GTTCGATAACTACGTCCTCGATGGTGTGGTACAGGCAGTGAGCGTTATTGAGGACATCACAGGCCAGGAACAAATCAACGCTGCGGGATATTGCATTGGTGGTACCGC
ACTTGCATCAGCGATTGCCTATTATGCCGCGAAACGTATGAAGAAACGCATCAAGAGTGCCAGCTTTTTTACAACGCTGCTTGACTTTAGCCAACCAGGTGAAGTGGGG
GCTTACATTAACGATACCATTATCTCCGCAATTGAAGCTCAGAATAGCGCCCAAGGCTTCATGGATGGGCGTTCGCTGTCAGTCACTTTTTCCCTGTTACGCGAGAATTC
CCTGTATTGGAACTATTACATTGACAACTATCTGAAAGGAACATCTCCGGTAGACTTTGACTTGCTTTACTGGAATTCGGATAGCACCAACGTTGCCGCAAACACCCAC
AATTTTCTGTTGCGTGAATTGTACCTGAACAACCAGTTAGTCCAGGACAAAGGCGTTAAGATTGGAGGCGTTTGGATTGATCTGAATAAAATCCGCATTCCGTCCTACT
TTATTAGCGCGAAAGACGATCACATAGCGCTGTGGCAGGGCACGTATCGAGGTGCTCTCGCAATGGGGGGCAATAAAACGTTTGTGCTCGGCGAATCGGGTCATATTG
CCGGGATTGTCAACCCACCTGCGAAAAACAAATACGGCTACTGGGTCAATGACAGTTTAGATGAATCGGCGGATGAATGGCTGGCTAATGCACAACGTGCGGAAGGTT
CATGGTGGACGCATTGGGACCAATGGCTGGACCAGTTCAATCCGGAATCCTTAGTACCGGCCTATCCGATTGGTTCGGACAACTTTCCGGCGTTAGAAGCGGCTCCCGG
CTCATATGTGAAACAGACCTTGCCGATTGTGGAATGA
